# Synthesis and crystal structure of NaRbB_5_O_8_(OH)·H_2_O

**DOI:** 10.1107/S2056989026007140

**Published:** 2026-07-21

**Authors:** Xin-Yue Zhang, Hui-Lin Xiao, Xiao-Liang Zhou, Mei-Jun Zou, Jian-Wen Cheng, Yi-Hang Wen

**Affiliations:** a Key Laboratory of the Ministry of Education for Advanced Catalysis Materials Institute of Physical Chemistry Zhejiang Normal University,Jinhua Zhejiang 321004 People’s Republic of China; University of Missouri-Columbia, USA

**Keywords:** alkali-metal borate, penta­borate unit, surfactant-thermal synthesis, hydrogen bond, crystal structure

## Abstract

A new mixed alkali-metal borate, poly[[sodium rubidium [hydroxido­octa-μ-oxido­penta­borate] monohydrate], NaRbB_5_O_8_(OH)·H_2_O, has been synthesized *via* a surfactant-thermal method using H_3_BO_3_, Rb_2_CO_3_, Na_2_SiO_3_·9H_2_O, ethanedi­amine and poly(ethyl­ene glycol)-400 as starting materials. It features a layered boron-oxide framework constructed from penta­borate [B_5_O_10_(OH)]^6−^ building units. Adjacent single layers are inter­connected to form double layers through hydrogen-bonding inter­actions. Na^+^, Rb^+^ cations and H_2_O mol­ecules are located in the voids of the framework.

## Abstract 2

In the title compound, the penta­borate [B_5_O_10_(OH)]^6−^ building units are inter­connected to form a two-dimensional layered structure, adjacent single layers are further linked into double layers *via* O—H⋯O hydrogen bonds.

## 1. Chemical context

Crystalline metal borates are widely recognized as promising non-linear optical materials in the ultraviolet region (Zhang *et al.*, 2026 ▸; Li *et al.*, 2023*a* ▸,*b* ▸,*c* ▸; Lu *et al.*, 2024 ▸; Tian *et al.*, 2013 ▸; Chen *et al.*, 2024*a* ▸, 2025 ▸; Wang *et al.*, 2017 ▸, 2025 ▸; Wei *et al.*, 2016 ▸; Yu *et al.*, 2017 ▸; Zhao *et al.*, 2025 ▸, 2026 ▸; Zou *et al.*, 2026*a* ▸). Boron can adopt either three- or four-coordinate geometries with oxygen atoms. Corner-sharing oxygen linkages between planar BO_3_ triangles and tetra­hedral BO_4_ units enable the construction of various polyborate anions (Mutailipu *et al.*, 2021 ▸), including [B_3_O_3_(OH)_4_]^−^, [B_4_O_5_(OH)_4_]^2−^, [B_7_O_12_(OH)_14_]^7−^, and [B_12_O_18_(OH)_6_]^6−^ (Lin *et al.*, 2011 ▸; Huang *et al.*, 2019 ▸; Chen *et al.*, 2024*c* ▸). Penta­borate units typically serve as fundamental building blocks. Various arrangements of BO_3_ triangles, BO_4_ tetra­hedra, as well as [B_5_O_*n*_] (*n*= 10–12, 14) and hy­droxy­lated [B_5_O_*n*_(OH)_*m*_] units, have been observed (Wei *et al.*, 2014 ▸; Ding *et al.*, 2018 ▸). These penta­borate anions are further inter­connected to construct chains, layers, and three-dimensional frameworks with rich structural diversity (Li *et al.*, 2024 ▸; Shi *et al.*, 2019 ▸; Zhao *et al.*, 2022 ▸, 2024 ▸; Chen *et al.*, 2024*b* ▸). In such crystal structures, alkali and alkaline-earth metal cations reside in inter­stitial voids to balance the overall charge of the polyborate anionic frameworks.
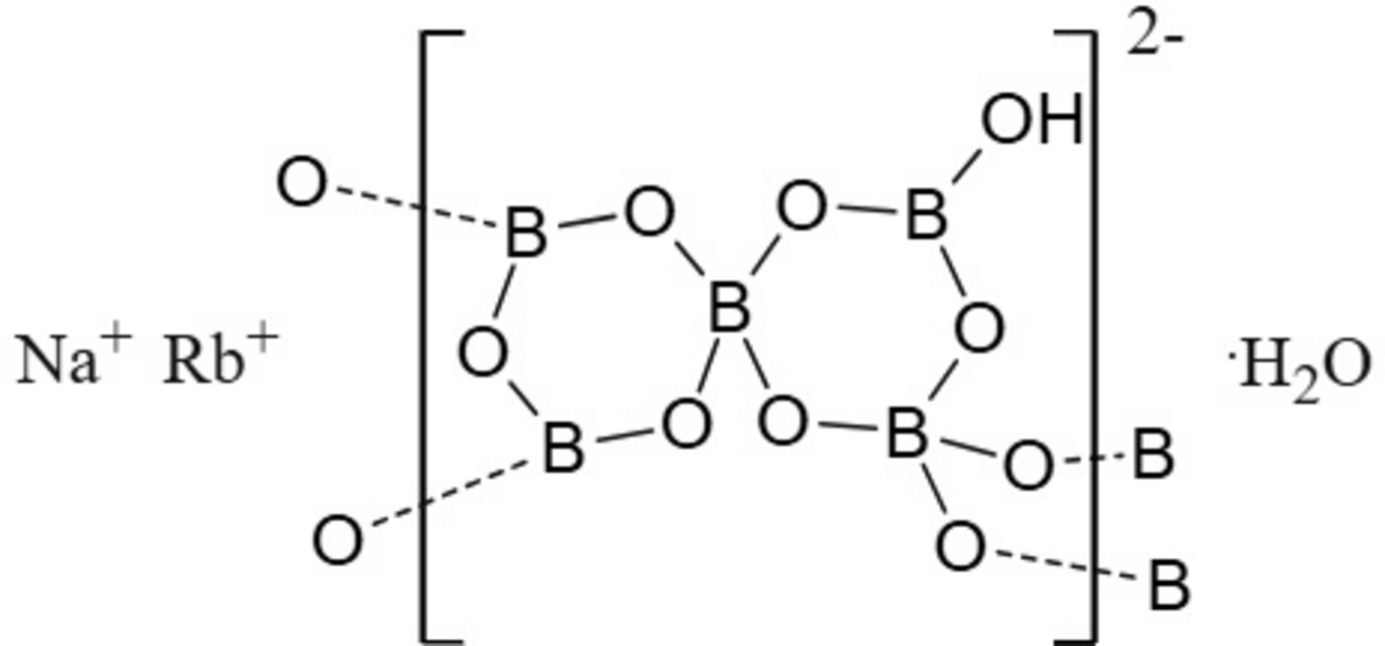


Borosilicates are new types of non-linear optical materials that combine different anionic groups (Ou *et al.*, 2025 ▸). Our initial synthetic strategy was aimed at the exploration of new borosilicate crystals. However, the targeted borosilicate phase was not obtained under the applied synthetic conditions, and a pure borate compound was ultimately isolated instead. Herein, we report the synthesis and single-crystal structural characterization of a new mixed alkali-metal penta­borate, NaRbB_5_O_8_(OH)·H_2_O, (I).

## 2. Structural commentary

**Figure 1:**
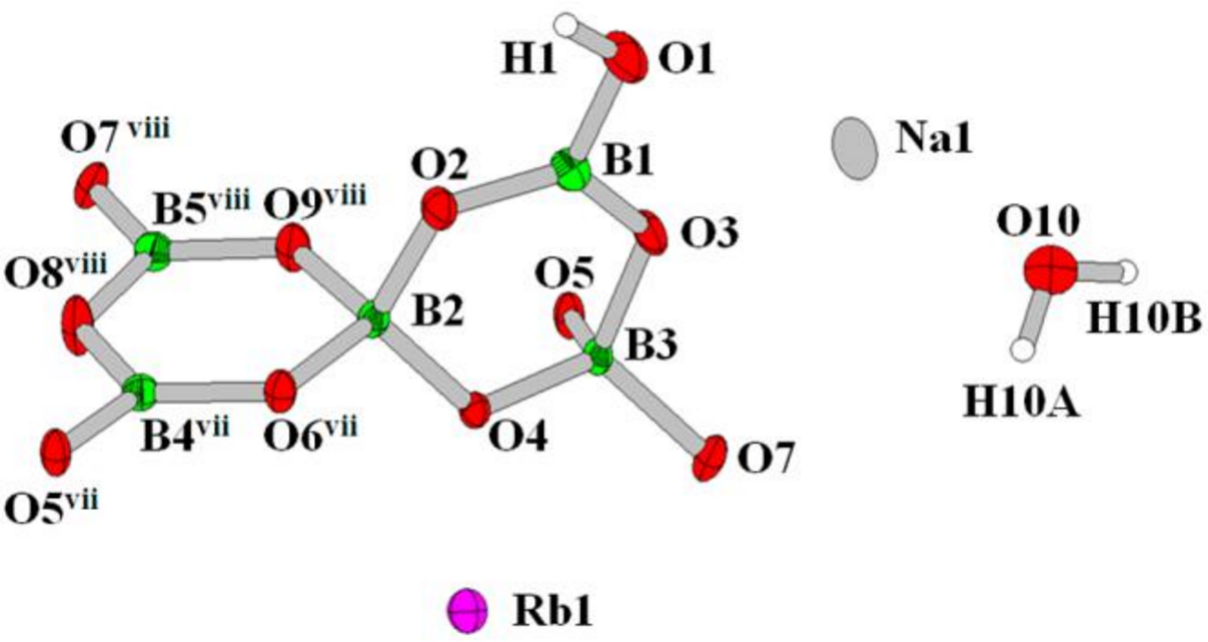
Structure of (I) with displacement ellipsoids drawn at the 50% probability level. [Symmetry codes: (vii) *x*, *y* − 1, *z*; (viii) *x* − 1, *y*, *z*].

**Figure 2:**
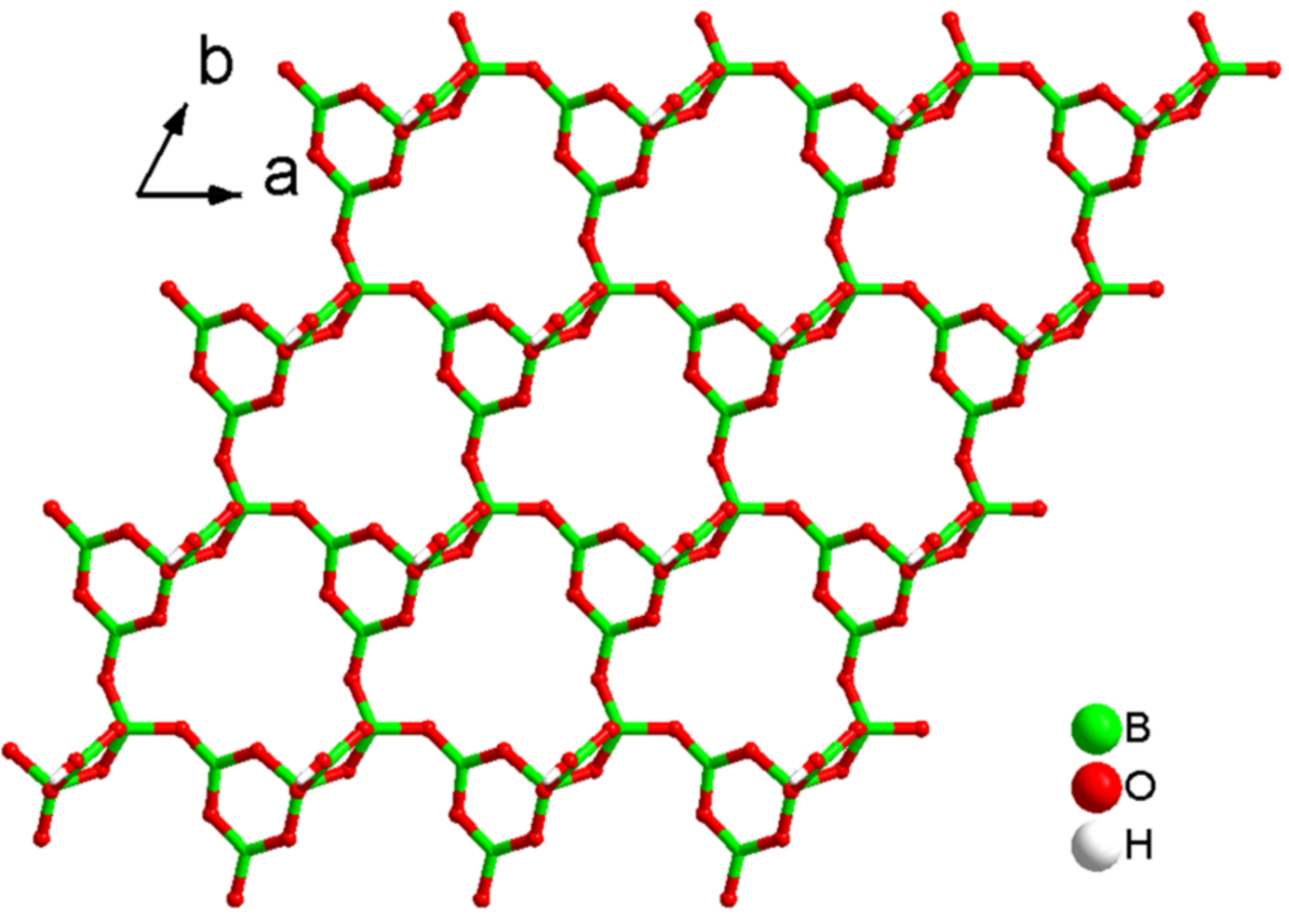
Two-dimensional layered structure of (I) in the *ab* plane.

The title compound crystallizes in the triclinic space group *P*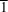. The asymmetric unit of (I) consists of five boron atoms, ten oxygen atoms, three hydrogen atoms, one Na^+^ cation and one Rb^+^ cation. As shown in Fig. 1 ▸, the fundamental building block of (I) is the [B_5_O_10_(OH)]^6−^ unit, which is composed of two [BO_3_] triangles, two [BO_4_] tetra­hedra and one [BO_2_(OH)] group. The B—O bond lengths fall in the range 1.344 (3)–1.506 (3) Å (Table 1 ▸). Each [B_5_O_10_(OH)]^6−^ unit links to four adjacent equivalents, generating a two-dimensional layered structure containing nine-membered ring windows in the *ab* plane (Fig. 2 ▸).

**Figure 3:**
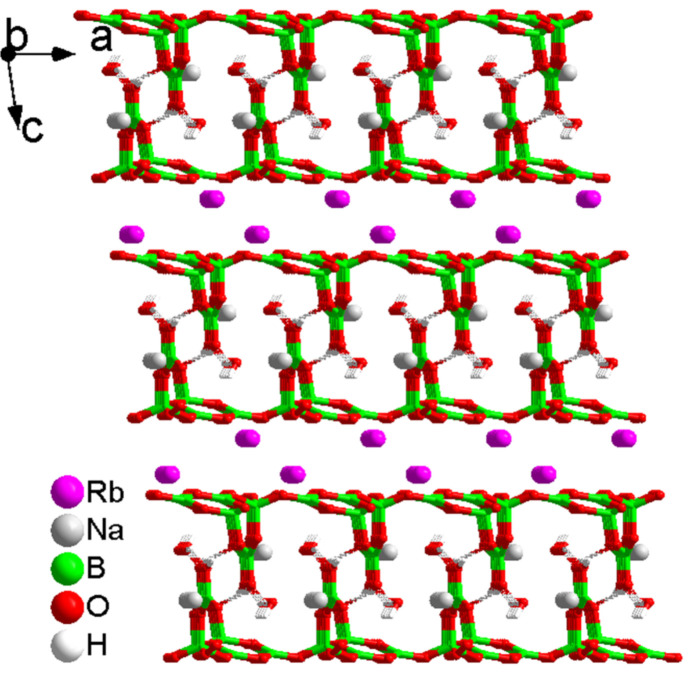
View of the three-dimensional packing structure of (I) along the *b* axis.

Adjacent single layers are connected into double layers through O—H⋯O hydrogen-bonding inter­actions between the hydroxyl group (O1) and oxygen atom (O2) of the [B_5_O_10_(OH)]^6−^ units. Hydrogen-bonding inter­actions also exist between the water mol­ecule (O10) and hydroxyl group (O1) in the anionic framework. The corresponding O⋯O hydrogen-bond distances range from 2.869 (3) to 2.916 (3) Å (Table 2 ▸). Its hydrogen-bonding inter­actions are highly analogous to those of the isotypic protonated borate NaKB_5_O_8_(OH)·H_2_O (Li *et al.*, 2024 ▸). These hydrogen-bonded double layers further stack parallel to one another along the *c* axis. Na^+^ ions and water mol­ecules reside in the inter­layer voids within the double layers, whereas Rb^+^ ions occupy the voids between adjacent double layers. The Na^+^ and Rb^+^ ions are six- and nine-coordinated, respectively, with Na—O distances of 2.320 (2)–2.773 (3) Å and Rb—O distances of 2.8044 (18)–3.1981 (19) Å (Table 1 ▸). These cations occupy the voids of the framework to maintain overall charge balance (Fig. 3 ▸).

## 3. Database survey

An online search of the Inorganic Crystal Structure Database (ICSD, version 5.6.0, updated January 2026, Zagorac *et al.*, 2019 ▸) for alkali-metal compounds incorporating the [B_5_O_10_(OH)]^6−^ moiety returned seven hits: NaKB_5_O_8_(OH)·H_2_O (triclinic, *P*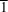 space group; Li *et al.*, 2024 ▸), LiRbB_5_O_8_(OH)·H_2_O (monoclinic, *P*2_1_/*n* space group; Shi *et al.*, 2019 ▸), K_2_B_5_O_8_(OH)·2H_2_O (ortho­rhom­bic, *Pna*2_1_ space group; Shi *et al.*, 2019 ▸), Rb_2_B_5_O_8_(OH) (ortho­rhom­bic, *Pca*2_1_ space group; Qiu *et al.*, 2021 ▸), LiCsB_5_O_8_(OH)·H_2_O (monoclinic, *P*2_1_/*c* space group; Chen *et al.*, 2017 ▸), LiKB_5_O_8_(OH)·1.5H_2_O (ortho­rhom­bic, *C*222_1_ space group; Li *et al.*, 2019 ▸), and Na_2_B_5_O_8_(OH)·2H_2_O (ortho­rhom­bic, *Pna*2_1_ space group; Wang *et al.*, 2009 ▸). In addition, we have recently reported another penta­borate, NaCsB_5_O_8_(OH)·H_2_O (ortho­rhom­bic, *Pbca* space group; Zou *et al.*, 2026*b* ▸). Compound (I) is isostructural with NaKB_5_O_8_(OH)·H_2_O, and they share the same space group and similar cell parameters. The other seven reported compounds share similar layered structural features and the common [B_5_O_10_(OH)]^6−^ building unit. Differences in alkali metal cations and space groups lead to the structural uniqueness of compound (I).

## 4. Synthesis and crystallization

A mixture of H_3_BO_3_ (0.6183 g, 10 mmol), Rb_2_CO_3_ (0.2308 g, 1 mmol), Na_2_SiO_3_·9H_2_O (0.2842 g, 1 mmol), ethanedi­amine (1 ml), and poly(ethyl­ene glycol)-400 (4 ml) was sealed in a 30 ml Teflon-lined bomb at 453 K for 6 days and then cooled to room temperature over 6 hours. Colorless crystals of (I) were obtained by filtration, washed with distilled water, and dried in air.

## 5. Refinement

Crystal data, data collection and structure refinement details are summarized in Table 3 ▸. H atoms bonded to O atoms were positioned geometrically and refined using a riding model [O_hydrox­yl_—H = 0.82 Å and O_water_—H = 0.84 Å, *U*_iso_(H) = 1.2 *U*_eq_(O)].

## Supplementary Material

Crystal structure: contains datablock(s) I, new. DOI: 10.1107/S2056989026007140/ev2027sup1.cif

Structure factors: contains datablock(s) I. DOI: 10.1107/S2056989026007140/ev2027Isup3.hkl

CCDC reference: 2543102

Additional supporting information:  crystallographic information; 3D view; checkCIF report

## Figures and Tables

**Table 1:** Selected bond lengths (Å)

Rb1—O8^i^	2.8044 (18)	B1—O2	1.365 (3)
Rb1—O6^ii^	2.8777 (18)	B1—O1	1.386 (4)
Rb1—O4	2.9174 (17)	B2—O4	1.420 (3)
Rb1—O7^ii^	2.9197 (17)	B2—O6^vii^	1.474 (3)
Rb1—O4^i^	2.9691 (17)	B2—O9^viii^	1.495 (3)
Rb1—O5	3.0402 (19)	B2—O2	1.504 (3)
Rb1—O9^ii^	3.0659 (19)	B3—O4	1.438 (3)
Rb1—O5^iii^	3.0669 (17)	B3—O7	1.469 (3)
Rb1—O7	3.1981 (19)	B3—O5	1.493 (3)
Na1—O3	2.320 (2)	B3—O3	1.506 (3)
Na1—O10	2.328 (2)	B4—O5	1.344 (3)
Na1—O6	2.372 (2)	B4—O6	1.364 (3)
Na1—O10^iv^	2.408 (3)	B4—O8^ix^	1.392 (3)
Na1—O2^v^	2.470 (2)	B5—O7	1.356 (3)
Na1—O1^vi^	2.773 (3)	B5—O9	1.359 (3)
B1—O3	1.348 (4)	B5—O8	1.383 (3)

Symmetry codes: (i) 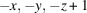; (ii) 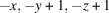; (iii) 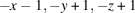; (iv) 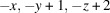; (v) 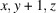; (vi) 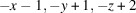; (vii) 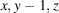; (viii) 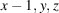; (ix) 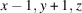.

**Table 2:** Hydrogen-bond geometry (Å, °)

*D*—H⋯*A*	*D*—H	H⋯*A*	*D*⋯*A*	*D*—H⋯*A*
O1—H1⋯O2^x^	0.82	2.11	2.869 (3)	155
O10—H10*B*⋯O1^iv^	0.84	2.16	2.916 (3)	151

Symmetry codes: (iv) 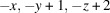; (x) 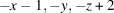.

**Table 3:** Experimental details

Crystal data	
Chemical formula	NaRbB_5_O_8_(OH)·H_2_O
*M*_r_	325.53
Crystal system, space group	Triclinic, *P* 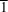
Temperature (K)	287
*a*, *b*, *c* (Å)	6.6580 (4), 6.6744 (4), 11.5322 (6)
α, β, γ (°)	79.094 (2), 77.508 (2), 60.492 (2)
*V* (Å^3^)	433.37 (4)
*Z*	2
Radiation type	Mo *K*α
μ (mm^−1^)	5.80
Crystal size (mm)	0.13 × 0.12 × 0.10

Data collection	
Diffractometer	Bruker APEXII area detector
Absorption correction	Empirical (using intensity measurements) (*SADABS*; Krause *et al.*, 2015 ▸)
*T*_min_, *T*_max_	0.48, 0.56
No. of measured, independent and observed [*I* > 2σ(*I*)] reflections	9111, 1985, 1863
*R*_int_	0.035
(sin θ/λ)_max_ (Å^−1^)	0.649

Refinement	
*R*[*F*^2^ > 2σ(*F*^2^)], *wR*(*F*^2^), *S*	0.026, 0.065, 1.08
No. of reflections	1985
No. of parameters	155
H-atom treatment	H-atom parameters constrained
Δρ_max_, Δρ_min_ (e Å^−3^)	0.87, −0.42

Computer programs: *APEX2* and *SAINT* (Bruker, 2014 ▸), *SHELXT* (Sheldrick, 2015*a* ▸), *SHELXL2014/7* (Sheldrick, 2015*b* ▸) and *OLEX2* (Dolomanov *et al.*, 2009 ▸).

## supplementary crystallographic information

### Poly[[sodium rubidium [hydroxidoocta-µ-oxidopentaborate] monohydrate] Crystal data

**Table:**

NaRbB_5_O_8_(OH)·H_2_O	*Z* = 2
*M**_r_* = 325.53	*F*(000) = 312
Triclinic, *P*1	*D*_x_ = 2.495 Mg m^−^^3^
*a* = 6.6580 (4) Å	Mo *K*α radiation, λ = 0.71073 Å
*b* = 6.6744 (4) Å	Cell parameters from 5543 reflections
*c* = 11.5322 (6) Å	θ = 3.5–27.5°
α = 79.094 (2)°	µ = 5.80 mm^−^^1^
β = 77.508 (2)°	*T* = 287 K
γ = 60.492 (2)°	Block, clear colourless
*V* = 433.37 (4) Å^3^	0.13 × 0.12 × 0.10 mm

### Poly[[sodium rubidium [hydroxidoocta-µ-oxidopentaborate] monohydrate] Data collection

**Table:**

Bruker APEXII area detector diffractometer	1863 reflections with *I* > 2σ(*I*)
ω scans	*R*_int_ = 0.035
Absorption correction: empirical (using intensity measurements) (SADABS; Krause *et al.*, 2015)	θ_max_ = 27.5°, θ_min_ = 3.5°
*T*_min_ = 0.48, *T*_max_ = 0.56	*h* = −8→8
9111 measured reflections	*k* = −8→8
1985 independent reflections	*l* = −14→14

### Poly[[sodium rubidium [hydroxidoocta-µ-oxidopentaborate] monohydrate] Refinement

**Table:**

Refinement on *F*^2^	Hydrogen site location: mixed
Least-squares matrix: full	H-atom parameters constrained
*R*[*F*^2^ > 2σ(*F*^2^)] = 0.026	*w* = 1/[σ^2^(*F*_o_^2^) + (0.0306*P*)^2^ + 0.5265*P*] where *P* = (*F*_o_^2^ + 2*F*_c_^2^)/3
*wR*(*F*^2^) = 0.065	(Δ/σ)_max_ < 0.001
*S* = 1.08	Δρ_max_ = 0.87 e Å^−^^3^
1985 reflections	Δρ_min_ = −0.42 e Å^−^^3^
155 parameters	Extinction correction: SHELXL2014/7 (Sheldrick 2015b), Fc^*^=kFc[1+0.001xFc^2^λ^3^/sin(2θ)]^-1/4^
0 restraints	Extinction coefficient: 0.005 (2)

### Poly[[sodium rubidium [hydroxidoocta-µ-oxidopentaborate] monohydrate] Special details

**Table:**

Geometry. All esds (except the esd in the dihedral angle between two l.s. planes) are estimated using the full covariance matrix. The cell esds are taken into account individually in the estimation of esds in distances, angles and torsion angles; correlations between esds in cell parameters are only used when they are defined by crystal symmetry. An approximate (isotropic) treatment of cell esds is used for estimating esds involving l.s. planes.

### Poly[[sodium rubidium [hydroxidoocta-µ-oxidopentaborate] monohydrate] Fractional atomic coordinates and isotropic or equivalent isotropic displacement parameters (Å^2^)

**Table:**

	*x*	*y*	*z*	*U*_iso_*/*U*_eq_	
Rb1	−0.15536 (4)	0.31899 (4)	0.42548 (2)	0.01816 (11)	
Na1	−0.1957 (2)	0.6023 (2)	0.90050 (11)	0.0262 (3)	
B1	−0.3018 (5)	0.1647 (5)	0.9121 (3)	0.0167 (6)	
B2	−0.3654 (5)	0.0470 (5)	0.7382 (3)	0.0126 (5)	
B3	−0.1935 (5)	0.3118 (4)	0.7072 (2)	0.0111 (5)	
B4	−0.4410 (5)	0.7519 (5)	0.6765 (3)	0.0133 (5)	
B5	0.2491 (5)	0.1475 (5)	0.6798 (3)	0.0147 (5)	
O1	−0.3247 (4)	0.1631 (4)	1.03462 (18)	0.0266 (5)	
H1	−0.3758	0.0744	1.0684	0.040*	
O2	−0.3634 (3)	0.0278 (3)	0.87005 (16)	0.0172 (4)	
O3	−0.2273 (3)	0.3071 (3)	0.84093 (16)	0.0182 (4)	
O4	−0.2045 (3)	0.1225 (3)	0.67186 (15)	0.0118 (3)	
O5	−0.3820 (3)	0.5345 (3)	0.66024 (17)	0.0152 (4)	
O6	−0.2980 (3)	0.8115 (3)	0.71344 (16)	0.0136 (4)	
O7	0.0306 (3)	0.3107 (3)	0.66079 (17)	0.0156 (4)	
O8	0.3356 (3)	−0.0786 (3)	0.6563 (2)	0.0228 (4)	
O9	0.3882 (3)	0.2091 (3)	0.71832 (18)	0.0182 (4)	
O10	0.1852 (4)	0.5348 (4)	0.8910 (2)	0.0294 (5)	
H10A	0.2601	0.4538	0.8343	0.044*	
H10B	0.2258	0.6376	0.8853	0.044*	

### Poly[[sodium rubidium [hydroxidoocta-µ-oxidopentaborate] monohydrate] Atomic displacement parameters (Å^2^)

**Table:**

	*U*^11^	*U*^22^	*U*^33^	*U*^12^	*U*^13^	*U*^23^
Rb1	0.01984 (15)	0.01750 (15)	0.02004 (15)	−0.01100 (11)	−0.00189 (9)	−0.00294 (9)
Na1	0.0329 (6)	0.0231 (6)	0.0287 (6)	−0.0154 (5)	−0.0134 (5)	0.0009 (5)
B1	0.0189 (14)	0.0152 (13)	0.0169 (14)	−0.0080 (11)	−0.0038 (11)	−0.0020 (11)
B2	0.0103 (12)	0.0104 (12)	0.0178 (14)	−0.0050 (10)	−0.0016 (10)	−0.0032 (10)
B3	0.0115 (12)	0.0082 (11)	0.0152 (13)	−0.0055 (10)	−0.0022 (10)	−0.0017 (10)
B4	0.0115 (12)	0.0122 (12)	0.0154 (13)	−0.0046 (10)	−0.0017 (10)	−0.0026 (10)
B5	0.0139 (13)	0.0120 (12)	0.0173 (14)	−0.0053 (11)	−0.0024 (10)	−0.0022 (10)
O1	0.0438 (13)	0.0338 (12)	0.0146 (9)	−0.0283 (10)	−0.0024 (9)	−0.0025 (8)
O2	0.0234 (9)	0.0200 (9)	0.0151 (9)	−0.0161 (8)	−0.0012 (7)	−0.0016 (7)
O3	0.0284 (10)	0.0197 (9)	0.0151 (9)	−0.0173 (8)	−0.0033 (8)	−0.0034 (7)
O4	0.0125 (8)	0.0102 (8)	0.0147 (8)	−0.0068 (7)	−0.0012 (6)	−0.0023 (6)
O5	0.0119 (8)	0.0094 (8)	0.0261 (10)	−0.0039 (7)	−0.0071 (7)	−0.0037 (7)
O6	0.0113 (8)	0.0086 (8)	0.0217 (9)	−0.0039 (7)	−0.0049 (7)	−0.0024 (7)
O7	0.0093 (8)	0.0107 (8)	0.0274 (10)	−0.0052 (7)	−0.0034 (7)	−0.0006 (7)
O8	0.0148 (9)	0.0124 (9)	0.0439 (13)	−0.0032 (7)	−0.0137 (8)	−0.0074 (8)
O9	0.0120 (9)	0.0107 (8)	0.0321 (11)	−0.0028 (7)	−0.0057 (7)	−0.0072 (7)
O10	0.0274 (11)	0.0318 (12)	0.0292 (12)	−0.0139 (9)	−0.0003 (9)	−0.0078 (9)

### Poly[[sodium rubidium [hydroxidoocta-µ-oxidopentaborate] monohydrate] Geometric parameters (Å, º)

**Table:**

Rb1—O8^i^	2.8044 (18)	B2—Rb1^i^	3.482 (3)
Rb1—O6^ii^	2.8777 (18)	B3—O4	1.438 (3)
Rb1—O4	2.9174 (17)	B3—O7	1.469 (3)
Rb1—O7^ii^	2.9197 (17)	B3—O5	1.493 (3)
Rb1—O4^i^	2.9691 (17)	B3—O3	1.506 (3)
Rb1—O5	3.0402 (19)	B4—O5	1.344 (3)
Rb1—O9^ii^	3.0659 (19)	B4—O6	1.364 (3)
Rb1—O5^iii^	3.0669 (17)	B4—O8^ix^	1.392 (3)
Rb1—B3	3.197 (3)	B4—Rb1^iii^	3.415 (3)
Rb1—O7	3.1981 (19)	B4—Rb1^ii^	3.703 (3)
Rb1—B5^ii^	3.323 (3)	B5—O7	1.356 (3)
Rb1—B4^iii^	3.415 (3)	B5—O9	1.359 (3)
Na1—O3	2.320 (2)	B5—O8	1.383 (3)
Na1—O10	2.328 (2)	B5—Rb1^ii^	3.323 (3)
Na1—O6	2.372 (2)	O1—Na1^vi^	2.773 (3)
Na1—O10^iv^	2.408 (3)	O1—H1	0.8200
Na1—O2^v^	2.470 (2)	O2—Na1^vii^	2.470 (2)
Na1—O1^vi^	2.773 (3)	O4—Rb1^i^	2.9691 (17)
Na1—B2^v^	3.017 (3)	O5—Rb1^iii^	3.0669 (17)
Na1—B4	3.095 (3)	O6—B2^v^	1.474 (3)
Na1—Na1^iv^	3.438 (2)	O6—Rb1^ii^	2.8778 (18)
Na1—Rb1^ii^	4.0661 (13)	O7—Rb1^ii^	2.9197 (17)
Na1—H10A	2.6638	O8—B4^x^	1.393 (3)
B1—O3	1.348 (4)	O8—Rb1^i^	2.8043 (18)
B1—O2	1.365 (3)	O9—B2^xi^	1.495 (3)
B1—O1	1.386 (4)	O9—Rb1^ii^	3.0659 (19)
B2—O4	1.420 (3)	O10—Na1^iv^	2.408 (3)
B2—O6^vii^	1.474 (3)	O10—Rb1^ii^	3.618 (2)
B2—O9^viii^	1.495 (3)	O10—H10A	0.8343
B2—O2	1.504 (3)	O10—H10B	0.8397
B2—Na1^vii^	3.017 (3)		
			
O8^i^—Rb1—O6^ii^	105.39 (5)	Na1^iv^—Na1—Rb1^ii^	106.24 (5)
O8^i^—Rb1—O4	93.14 (6)	O3—Na1—H10A	99.4
O6^ii^—Rb1—O4	120.87 (5)	O10—Na1—H10A	17.6
O8^i^—Rb1—O7^ii^	139.14 (6)	O6—Na1—H10A	94.4
O6^ii^—Rb1—O7^ii^	63.54 (5)	O10^iv^—Na1—H10A	97.8
O4—Rb1—O7^ii^	126.94 (5)	O2^v^—Na1—H10A	103.5
O8^i^—Rb1—O4^i^	65.68 (5)	O1^vi^—Na1—H10A	169.4
O6^ii^—Rb1—O4^i^	48.12 (5)	B2^v^—Na1—H10A	97.7
O4—Rb1—O4^i^	96.20 (4)	B4—Na1—H10A	109.6
O7^ii^—Rb1—O4^i^	111.32 (5)	Na1^iv^—Na1—H10A	56.6
O8^i^—Rb1—O5	119.37 (6)	Rb1^ii^—Na1—H10A	50.7
O6^ii^—Rb1—O5	132.86 (5)	O3—B1—O2	123.4 (2)
O4—Rb1—O5	47.21 (5)	O3—B1—O1	118.3 (2)
O7^ii^—Rb1—O5	88.93 (5)	O2—B1—O1	118.2 (2)
O4^i^—Rb1—O5	141.35 (5)	O4—B2—O6^vii^	111.0 (2)
O8^i^—Rb1—O9^ii^	100.57 (5)	O4—B2—O9^viii^	113.1 (2)
O6^ii^—Rb1—O9^ii^	94.20 (5)	O6^vii^—B2—O9^viii^	110.6 (2)
O4—Rb1—O9^ii^	137.30 (5)	O4—B2—O2	111.0 (2)
O7^ii^—Rb1—O9^ii^	46.02 (5)	O6^vii^—B2—O2	104.7 (2)
O4^i^—Rb1—O9^ii^	126.34 (5)	O9^viii^—B2—O2	106.1 (2)
O5—Rb1—O9^ii^	91.62 (5)	O4—B2—Na1^vii^	120.06 (16)
O8^i^—Rb1—O5^iii^	46.44 (5)	O6^vii^—B2—Na1^vii^	50.58 (11)
O6^ii^—Rb1—O5^iii^	128.56 (5)	O9^viii^—B2—Na1^vii^	126.80 (17)
O4—Rb1—O5^iii^	104.92 (5)	O2—B2—Na1^vii^	54.58 (11)
O7^ii^—Rb1—O5^iii^	107.34 (5)	O4—B2—Rb1^i^	57.43 (11)
O4^i^—Rb1—O5^iii^	108.89 (5)	O6^vii^—B2—Rb1^i^	54.13 (11)
O5—Rb1—O5^iii^	94.80 (5)	O9^viii^—B2—Rb1^i^	137.87 (17)
O9^ii^—Rb1—O5^iii^	61.34 (5)	O2—B2—Rb1^i^	115.65 (15)
O8^i^—Rb1—B3	118.10 (7)	Na1^vii^—B2—Rb1^i^	77.10 (6)
O6^ii^—Rb1—B3	117.20 (6)	O4—B3—O7	112.4 (2)
O4—Rb1—B3	26.71 (6)	O4—B3—O5	109.14 (19)
O7^ii^—Rb1—B3	100.64 (6)	O7—B3—O5	107.95 (19)
O4^i^—Rb1—B3	113.99 (6)	O4—B3—O3	111.7 (2)
O5—Rb1—B3	27.55 (6)	O7—B3—O3	107.32 (19)
O9^ii^—Rb1—B3	117.66 (6)	O5—B3—O3	108.17 (19)
O5^iii^—Rb1—B3	114.24 (6)	O4—B3—Rb1	65.75 (12)
O8^i^—Rb1—O7	138.07 (5)	O7—B3—Rb1	76.77 (13)
O6^ii^—Rb1—O7	91.65 (5)	O5—B3—Rb1	70.37 (12)
O4—Rb1—O7	46.24 (4)	O3—B3—Rb1	175.90 (16)
O7^ii^—Rb1—O7	82.76 (5)	O5—B4—O6	123.9 (2)
O4^i^—Rb1—O7	103.24 (5)	O5—B4—O8^ix^	116.5 (2)
O5—Rb1—O7	45.07 (4)	O6—B4—O8^ix^	119.6 (2)
O9^ii^—Rb1—O7	116.34 (5)	O5—B4—Na1	93.79 (16)
O5^iii^—Rb1—O7	139.24 (5)	O6—B4—Na1	46.49 (12)
B3—Rb1—O7	26.56 (6)	O8^ix^—B4—Na1	131.13 (18)
O8^i^—Rb1—B5^ii^	124.32 (6)	O5—B4—Rb1^iii^	63.74 (13)
O6^ii^—Rb1—B5^ii^	83.61 (6)	O6—B4—Rb1^iii^	172.23 (18)
O4—Rb1—B5^ii^	129.51 (6)	O8^ix^—B4—Rb1^iii^	52.91 (12)
O7^ii^—Rb1—B5^ii^	23.98 (6)	Na1—B4—Rb1^iii^	138.69 (10)
O4^i^—Rb1—B5^ii^	127.99 (6)	O5—B4—Rb1^ii^	94.43 (15)
O5—Rb1—B5^ii^	83.11 (6)	O6—B4—Rb1^ii^	43.80 (12)
O9^ii^—Rb1—B5^ii^	24.12 (6)	O8^ix^—B4—Rb1^ii^	135.22 (17)
O5^iii^—Rb1—B5^ii^	84.63 (6)	Na1—B4—Rb1^ii^	72.86 (6)
B3—Rb1—B5^ii^	103.94 (7)	Rb1^iii^—B4—Rb1^ii^	138.55 (9)
O7—Rb1—B5^ii^	95.01 (6)	O7—B5—O9	119.4 (2)
O8^i^—Rb1—B4^iii^	23.33 (6)	O7—B5—O8	121.0 (2)
O6^ii^—Rb1—B4^iii^	119.67 (6)	O9—B5—O8	119.5 (2)
O4—Rb1—B4^iii^	99.01 (6)	O7—B5—Rb1^ii^	61.07 (13)
O7^ii^—Rb1—B4^iii^	125.52 (6)	O9—B5—Rb1^ii^	67.26 (14)
O4^i^—Rb1—B4^iii^	87.64 (6)	O8—B5—Rb1^ii^	147.58 (19)
O5—Rb1—B4^iii^	107.45 (6)	B1—O1—Na1^vi^	102.67 (17)
O9^ii^—Rb1—B4^iii^	81.07 (6)	B1—O1—H1	109.5
O5^iii^—Rb1—B4^iii^	23.13 (6)	Na1^vi^—O1—H1	68.7
B3—Rb1—B4^iii^	117.84 (7)	B1—O2—B2	118.9 (2)
O7—Rb1—B4^iii^	143.96 (6)	B1—O2—Na1^vii^	128.30 (17)
B5^ii^—Rb1—B4^iii^	105.15 (7)	B2—O2—Na1^vii^	95.68 (14)
O3—Na1—O10	114.72 (9)	B1—O3—B3	120.6 (2)
O3—Na1—O6	87.80 (7)	B1—O3—Na1	126.20 (17)
O10—Na1—O6	102.94 (8)	B3—O3—Na1	112.48 (14)
O3—Na1—O10^iv^	97.54 (8)	B2—O4—B3	120.1 (2)
O10—Na1—O10^iv^	86.93 (9)	B2—O4—Rb1	132.21 (15)
O6—Na1—O10^iv^	165.69 (9)	B3—O4—Rb1	87.55 (13)
O3—Na1—O2^v^	139.94 (8)	B2—O4—Rb1^i^	98.80 (13)
O10—Na1—O2^v^	94.71 (8)	B3—O4—Rb1^i^	133.22 (14)
O6—Na1—O2^v^	58.24 (6)	Rb1—O4—Rb1^i^	83.80 (4)
O10^iv^—Na1—O2^v^	111.23 (8)	B4—O5—B3	129.0 (2)
O3—Na1—O1^vi^	90.30 (8)	B4—O5—Rb1	124.85 (16)
O10—Na1—O1^vi^	154.55 (9)	B3—O5—Rb1	82.08 (13)
O6—Na1—O1^vi^	81.58 (7)	B4—O5—Rb1^iii^	93.13 (14)
O10^iv^—Na1—O1^vi^	85.11 (8)	B3—O5—Rb1^iii^	135.15 (14)
O2^v^—Na1—O1^vi^	66.05 (7)	Rb1—O5—Rb1^iii^	85.20 (5)
O3—Na1—B2^v^	115.13 (8)	B4—O6—B2^v^	124.4 (2)
O10—Na1—B2^v^	97.70 (9)	B4—O6—Na1	108.88 (16)
O6—Na1—B2^v^	28.69 (7)	B2^v^—O6—Na1	100.73 (14)
O10^iv^—Na1—B2^v^	140.75 (9)	B4—O6—Rb1^ii^	117.06 (15)
O2^v^—Na1—B2^v^	29.74 (7)	B2^v^—O6—Rb1^ii^	101.35 (13)
O1^vi^—Na1—B2^v^	74.11 (7)	Na1—O6—Rb1^ii^	101.09 (6)
O3—Na1—B4	66.83 (7)	B5—O7—B3	129.6 (2)
O10—Na1—B4	122.57 (9)	B5—O7—Rb1^ii^	94.94 (15)
O6—Na1—B4	24.64 (7)	B3—O7—Rb1^ii^	132.55 (14)
O10^iv^—Na1—B4	150.00 (9)	B5—O7—Rb1	119.02 (16)
O2^v^—Na1—B4	74.63 (7)	B3—O7—Rb1	76.67 (13)
O1^vi^—Na1—B4	70.18 (7)	Rb1^ii^—O7—Rb1	97.24 (5)
B2^v^—Na1—B4	48.48 (8)	B5—O8—B4^x^	121.5 (2)
O3—Na1—Na1^iv^	112.03 (7)	B5—O8—Rb1^i^	134.67 (16)
O10—Na1—Na1^iv^	44.38 (6)	B4^x^—O8—Rb1^i^	103.76 (15)
O6—Na1—Na1^iv^	146.14 (8)	B5—O9—B2^xi^	124.3 (2)
O10^iv^—Na1—Na1^iv^	42.55 (6)	B5—O9—Rb1^ii^	88.62 (15)
O2^v^—Na1—Na1^iv^	108.01 (7)	B2^xi^—O9—Rb1^ii^	133.40 (15)
O1^vi^—Na1—Na1^iv^	123.50 (7)	Na1—O10—Na1^iv^	93.07 (9)
B2^v^—Na1—Na1^iv^	129.28 (8)	Na1—O10—Rb1^ii^	83.25 (7)
B4—Na1—Na1^iv^	166.20 (8)	Na1^iv^—O10—Rb1^ii^	172.67 (9)
O3—Na1—Rb1^ii^	90.67 (6)	Na1—O10—H10A	104.6
O10—Na1—Rb1^ii^	62.09 (6)	Na1^iv^—O10—H10A	125.6
O6—Na1—Rb1^ii^	43.99 (5)	Rb1^ii^—O10—H10A	50.0
O10^iv^—Na1—Rb1^ii^	148.42 (7)	Na1—O10—H10B	125.3
O2^v^—Na1—Rb1^ii^	79.34 (5)	Na1^iv^—O10—H10B	101.5
O1^vi^—Na1—Rb1^ii^	125.46 (6)	Rb1^ii^—O10—H10B	85.8
B2^v^—Na1—Rb1^ii^	56.58 (6)	H10A—O10—H10B	108.3
B4—Na1—Rb1^ii^	60.48 (6)		
			
O3—B1—O1—Na1^vi^	106.2 (2)	Rb1^iii^—B4—O5—Rb1	86.33 (13)
O2—B1—O1—Na1^vi^	−71.6 (3)	Rb1^ii^—B4—O5—Rb1	−56.82 (15)
O3—B1—O2—B2	−4.5 (4)	O6—B4—O5—Rb1^iii^	−178.1 (2)
O1—B1—O2—B2	173.2 (2)	O8^ix^—B4—O5—Rb1^iii^	3.4 (2)
O3—B1—O2—Na1^vii^	121.0 (2)	Na1—B4—O5—Rb1^iii^	143.78 (7)
O1—B1—O2—Na1^vii^	−61.3 (3)	Rb1^ii^—B4—O5—Rb1^iii^	−143.15 (6)
O4—B2—O2—B1	27.4 (3)	O4—B3—O5—B4	176.8 (2)
O6^vii^—B2—O2—B1	147.2 (2)	O7—B3—O5—B4	−60.8 (3)
O9^viii^—B2—O2—B1	−95.9 (3)	O3—B3—O5—B4	55.0 (3)
Na1^vii^—B2—O2—B1	140.1 (2)	Rb1—B3—O5—B4	−129.0 (2)
Rb1^i^—B2—O2—B1	90.3 (2)	O4—B3—O5—Rb1	−54.17 (17)
O4—B2—O2—Na1^vii^	−112.69 (17)	O7—B3—O5—Rb1	68.22 (16)
O6^vii^—B2—O2—Na1^vii^	7.12 (18)	O3—B3—O5—Rb1	−175.95 (17)
O9^viii^—B2—O2—Na1^vii^	124.07 (16)	O4—B3—O5—Rb1^iii^	20.8 (3)
Rb1^i^—B2—O2—Na1^vii^	−49.79 (13)	O7—B3—O5—Rb1^iii^	143.17 (16)
O2—B1—O3—B3	−3.8 (4)	O3—B3—O5—Rb1^iii^	−101.0 (2)
O1—B1—O3—B3	178.5 (2)	Rb1—B3—O5—Rb1^iii^	74.95 (16)
O2—B1—O3—Na1	165.68 (19)	O5—B4—O6—B2^v^	−176.3 (2)
O1—B1—O3—Na1	−12.0 (4)	O8^ix^—B4—O6—B2^v^	2.1 (4)
O4—B3—O3—B1	−11.5 (3)	Na1—B4—O6—B2^v^	−118.2 (3)
O7—B3—O3—B1	−135.1 (2)	Rb1^ii^—B4—O6—B2^v^	128.1 (3)
O5—B3—O3—B1	108.7 (2)	O5—B4—O6—Na1	−58.2 (3)
O4—B3—O3—Na1	177.71 (15)	O8^ix^—B4—O6—Na1	120.3 (2)
O7—B3—O3—Na1	54.1 (2)	Rb1^ii^—B4—O6—Na1	−113.76 (15)
O5—B3—O3—Na1	−62.1 (2)	O5—B4—O6—Rb1^ii^	55.6 (3)
O6^vii^—B2—O4—B3	−161.2 (2)	O8^ix^—B4—O6—Rb1^ii^	−126.0 (2)
O9^viii^—B2—O4—B3	73.8 (3)	Na1—B4—O6—Rb1^ii^	113.76 (15)
O2—B2—O4—B3	−45.2 (3)	O9—B5—O7—B3	−127.1 (3)
Na1^vii^—B2—O4—B3	−105.5 (2)	O8—B5—O7—B3	55.3 (4)
Rb1^i^—B2—O4—B3	−153.0 (2)	Rb1^ii^—B5—O7—B3	−162.2 (3)
O6^vii^—B2—O4—Rb1	81.4 (2)	O9—B5—O7—Rb1^ii^	35.1 (3)
O9^viii^—B2—O4—Rb1	−43.6 (3)	O8—B5—O7—Rb1^ii^	−142.5 (2)
O2—B2—O4—Rb1	−162.63 (13)	O9—B5—O7—Rb1	136.2 (2)
Na1^vii^—B2—O4—Rb1	137.07 (12)	O8—B5—O7—Rb1	−41.4 (3)
Rb1^i^—B2—O4—Rb1	89.59 (15)	Rb1^ii^—B5—O7—Rb1	101.11 (12)
O6^vii^—B2—O4—Rb1^i^	−8.2 (2)	O4—B3—O7—B5	−60.4 (3)
O9^viii^—B2—O4—Rb1^i^	−133.16 (18)	O5—B3—O7—B5	179.2 (2)
O2—B2—O4—Rb1^i^	107.78 (17)	O3—B3—O7—B5	62.8 (3)
Na1^vii^—B2—O4—Rb1^i^	47.47 (16)	Rb1—B3—O7—B5	−116.8 (2)
O7—B3—O4—B2	158.4 (2)	O4—B3—O7—Rb1^ii^	144.03 (15)
O5—B3—O4—B2	−81.9 (3)	O5—B3—O7—Rb1^ii^	23.6 (3)
O3—B3—O4—B2	37.6 (3)	O3—B3—O7—Rb1^ii^	−92.7 (2)
Rb1—B3—O4—B2	−138.8 (2)	Rb1—B3—O7—Rb1^ii^	87.61 (16)
O7—B3—O4—Rb1	−62.82 (18)	O4—B3—O7—Rb1	56.42 (17)
O5—B3—O4—Rb1	56.89 (17)	O5—B3—O7—Rb1	−63.97 (16)
O3—B3—O4—Rb1	176.48 (17)	O3—B3—O7—Rb1	179.65 (17)
O7—B3—O4—Rb1^i^	16.3 (3)	O7—B5—O8—B4^x^	−179.4 (2)
O5—B3—O4—Rb1^i^	136.02 (16)	O9—B5—O8—B4^x^	3.0 (4)
O3—B3—O4—Rb1^i^	−104.4 (2)	Rb1^ii^—B5—O8—B4^x^	96.7 (4)
Rb1—B3—O4—Rb1^i^	79.14 (15)	O7—B5—O8—Rb1^i^	−1.6 (4)
O6—B4—O5—B3	18.6 (4)	O9—B5—O8—Rb1^i^	−179.15 (17)
O8^ix^—B4—O5—B3	−159.9 (2)	Rb1^ii^—B5—O8—Rb1^i^	−85.5 (4)
Na1—B4—O5—B3	−19.5 (3)	O7—B5—O9—B2^xi^	−177.7 (2)
Rb1^iii^—B4—O5—B3	−163.3 (3)	O8—B5—O9—B2^xi^	−0.1 (4)
Rb1^ii^—B4—O5—B3	53.5 (3)	Rb1^ii^—B5—O9—B2^xi^	−144.7 (2)
O6—B4—O5—Rb1	−91.7 (3)	O7—B5—O9—Rb1^ii^	−33.1 (2)
O8^ix^—B4—O5—Rb1	89.8 (3)	O8—B5—O9—Rb1^ii^	144.5 (2)
Na1—B4—O5—Rb1	−129.89 (11)		

Symmetry codes: (i) −*x*, −*y*, −*z*+1; (ii) −*x*, −*y*+1, −*z*+1; (iii) −*x*−1, −*y*+1, −*z*+1; (iv) −*x*, −*y*+1, −*z*+2; (v) *x*, *y*+1, *z*; (vi) −*x*−1, −*y*+1, −*z*+2; (vii) *x*, *y*−1, *z*; (viii) *x*−1, *y*, *z*; (ix) *x*−1, *y*+1, *z*; (x) *x*+1, *y*−1, *z*; (xi) *x*+1, *y*, *z*.

### Poly[[sodium rubidium [hydroxidoocta-µ-oxidopentaborate] monohydrate] Hydrogen-bond geometry (Å, º)

**Table:**

*D*—H···*A*	*D*—H	H···*A*	*D*···*A*	*D*—H···*A*
O1—H1···O2^xii^	0.82	2.11	2.869 (3)	155
O10—H10*B*···O1^iv^	0.84	2.16	2.916 (3)	151

Symmetry codes: (iv) −*x*, −*y*+1, −*z*+2; (xii) −*x*−1, −*y*, −*z*+2.
